# Analgesic and Anti-Inflammatory Effects of the Synthetic Neurosteroid Analogue BNN27 during CFA-Induced Hyperalgesia

**DOI:** 10.3390/biomedicines9091185

**Published:** 2021-09-09

**Authors:** Smaragda Poulaki, Olga Rassouli, George Liapakis, Achille Gravanis, Maria Venihaki

**Affiliations:** 1 Department of Clinical Chemistry, Medical School, University of Crete, Voutes, 71110 Heraklion, Greece; s.poulaki@med.uoc.gr (S.P.); orassouli@edu.med.uoc.gr (O.R.); 2 Department of Pharmacology, Medical School, University of Crete, Voutes, 71110 Heraklion, Greece; liapakis@med.uoc.gr (G.L.); gravanis@med.uoc.gr (A.G.); 3 Institute of Molecular Biology & Biotechnology, Foundation of Research & Technology-Hellas, 71110 Heraklion, Greece

**Keywords:** hyperalgesia, inflammation, neurosteroids, cytokines, NGF, opioids

## Abstract

Dehydroepiandrosterone (DHEA), an adrenal and neurosteroid hormone with strong neuroprotective and immunomodulatory properties, and ligand for all high-affinity neurotrophin tyrosine kinase receptors (Trk), also exerts important effects on hyperalgesia. Its synthetic, 17-spiro-epoxy analogue, BNN27, cannot be converted to estrogen or androgen as DHEA; it is a specific agonist of TrkA, the receptor of pain regulator Nerve Growth Factor (NGF), and it conserves the immunomodulatory properties of DHEA. Our study aimed to evaluate the anti-nociceptive and anti-inflammatory properties of BNN27 during Complete Freund’s Adjuvant (CFA)-induced inflammatory hyperalgesia in mice. Hyperalgesia was evaluated using the Hargreaves test. Inflammatory markers such as cytokines, NGF and opioids were measured, additionally to corticosterone and the protein kinase B (AKT) signaling pathway. We showed for the first time that treatment with BNN27 reversed hyperalgesia produced by CFA. The effect of BNN27 involved the inhibition of NGF in the dorsal root ganglia (DRG) and the increased synthesis of opioid peptides and their receptors in the inflamed paw. We also found alterations in the cytokine levels as well as in the phosphorylation of AKT2. Our findings strongly support that BNN27 represents a lead molecule for the development of analgesic and anti-inflammatory compounds with potential therapeutic applications in inflammatory hyperalgesia.

## 1. Introduction

Tissue injury or infection results in inflammation, characterized by hyperalgesia hypersensitivity as well as activation of the immune system and promotion of the healing processes of the tissue [1]. Indeed, during the inflammatory process, immune cells synthesize and release various inflammatory mediators, such as cytokines, neuropeptides and nitric oxide (NO) [2,3], further enhancing inflammation and sensitizing nociceptors in the peripheral terminals of primary sensory neurons. In addition, these agents alter vascular permeability and blood flow, and activate the immune cells to migrate at the site of inflammation, producing more chemical mediators [4,5]. 

Accumulating evidence indicates the relationship between cytokines and inflammatory hyperalgesia [6]. Thus, administration of interleukin-6 (IL-6) leads to hyperalgesia, while IL-6 knockout mice exhibit decreased heat nociception [7,8]. Similar effects have been described for tumor necrosis factor-alpha (TNF-α) and interleukin-1 beta (IL-1β). Indeed, intraperitoneal (i.p) administration of TNF-α in rats produces hyperalgesia, which is mediated by IL-1β in a dose-dependent manner, while its blockade reduces lipopolysaccharide (LPS) hyperalgesia in rats [9,10]. A more recent study supporting the involvement of TNF-α in hyperalgesia showed that in rats with peripheral neuropathy, TNF-α blockade alleviates hyperalgesia by decreasing the p 38 mitogen-activated protein kinase (p38-MAP) and c-Jun N-terminal kinase (JNK) signaling pathways in dorsal root ganglion (DRG) [11]. Interleukin IL-1β is also a key factor in the process of hyperalgesia. Thus, its administration was shown to induce hyperalgesia in rats [10] and, in synchrony with TNF-α and IL-6, sensitizes rat paw nociceptors to heat; it also controls the release of calcitonin gene-related peptide (CGRP), a local factor associated to heat hyperalgesia [8]. Lately, Hao and his co-workers provided evidence that reduced expression of IL-1β and other inflammatory mediators, such as inducible nitric oxide (iNOS), attenuated visceral hyperalgesia in rats [12]. 

The secretion of cytokines also has been correlated with other hyperalgesia and analgesia mediators. For instance, intra-plantar administration of IL-1β is linked to enhanced axonal transport of substance P and μ and κ opioid receptors in sensory neurons in rats [13]. Anti-inflammatory cytokines also have a key role in the inflammatory hyperalgesia response. Actually, interleukin-10 (IL-10) seems to be an important contributor in hyperalgesia since low mRNA levels of IL-10 were shown in patients with chronic pain [14]. In addition to inflammatory cytokines, opioid peptides are also main players in the mechanisms of inflammation-induced analgesia [15]. Opioid-mediated analgesia is extensively studied not only in the central nervous system (CNS) but also in the periphery, since immune cells have been shown to produce and release opioid peptides (β-endorphin and encephalin) and express opioid receptors at the site of the inflammation [16,17,18].

The steroid dehydroepiandrosterone (DHEA), produced by the adrenals but also by central and peripheral neurons [19,20], has been shown to possess analgesic and anti-inflammatory effects. Indeed, there is evidence that DHEA modulates pain signal transmission to the brain [21]. Low DHEA levels are also involved in the inflammatory hyperalgesia, as it has been shown that its levels were reduced in the spinal cord of rats after carrageenan-induced inflammation [22]. Previous studies demonstrated that DHEA could exert its effects by binding, among others, to NGF receptors TrkA and pan neurotrophin receptor (p75NTR), mimicking its neurotrophic actions [23]. In fact, this fascinating neurosteroid has the ability to bind to almost all neurotrophin receptors, including tyrosine kinase receptor B (TrkB) and TrkC families [24]. Recently, DHEA was shown to contribute to neuroinflammatory microglia regulation via TrkA receptors and the downstream AKT1/AKT2 signaling pathway in mice [25]. It is of note that neurotrophin NGF and its receptor TrkA contribute to hyperalgesia. Indeed, NGF was shown to rapidly increase at the site of inflammation [26]. Additionally, the levels of NGF were found elevated in the red nucleus (RN) of rats with spared nerve injury (SNI), while its blockade resulted in reduced mechanical allodynia [27]. Furthermore, administration of anti-TrkA monoclonal antibodies suppressed not only inflammatory but also neuropathic pain in mice [28], while inhibition of TrkA in the hind paw of rats prevented thermal hyperalgesia, produced by NGF administration [29]. 

**Figure 1:**
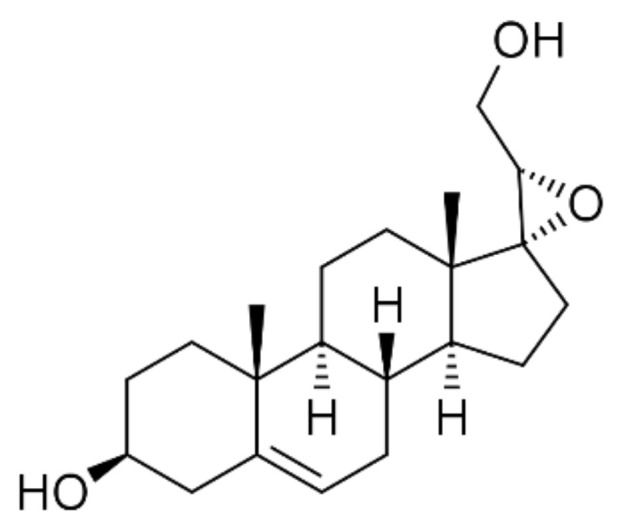
Chemical structure of 17alpha, 20R-epoxypregn-5-ene-3β, 21-diol (BNN27).

The novel 17-spiro-epoxy synthetic derivative of DHEA, BNN27 (17alpha, 20*R*-epoxypregn-5-ene-eβ, 21-diol, MW 332, 480; Figure 1), binds specifically to NGF receptor TrkA, showing no affinity for the other neurotrophin receptors, and exerting strong prosurvival and anti-apoptotic effects in various types of neurons [30]. This compound is also deprived of DHEA endocrine effects and the ability of the latter to rapidly metabolize to androgens and estrogens, associated with an increased risk of hormone-dependent tumors [31]. Recently, BNN27 was shown to protect the retina of diabetic rats in a TrkA-mediated manner, decreasing the activation of microglia and the production of pro-inflammatory TNF-alpha and IL-6, while strongly inducing the anti-inflammatory cytokines interleukin-4 (IL-4) and IL-10 [32]. In addition, BNN27 prevented, TrkA-dependently, the apoptosis of oligodendrocytes and microglia in mice, challenged with cuprizone, a demyelinating neurotoxin [33]. 

In the present study, we assessed the effects of BNN27 and its underlying mechanism of action in inflammatory hyperalgesia, using one of the most established experimental models of this condition, the intra-plantar injection of Complete Freund’s Adjuvant (CFA) into the mouse hind paw, which leads to thermal hyperalgesia and an excessive local and systemic inflammatory response [34,35]. Our findings suggest that BNN27 can effectively reduce thermal hyperalgesia in mice, inhibiting the synthesis of hyperalgesic NGF in dorsal root ganglion (DRG) and increasing the levels of opioid peptides and their receptors at the site of inflammation. These actions are mediated by the TrkA receptor and the downstream inhibition of the AKT2 signaling pathway. Thus, our data support the hypothesis that BNN27 may represent a lead molecule to develop new therapeutic agents against inflammatory hyperalgesia.

## 2. Materials and Methods

### 2.1. Animals

Experiments were carried out in adult (8–12 weeks) male mice of C57BL6x1291Sv genetic background. All animals were generated by in-house breeding. Mice were maintained on a 12:12 h light:dark schedule and room temperature at 22 ± 2 °C, with food and water ad libitum. Experiments and animal care had been approved by the Committee of Experimental Animal Care and Protocols of the University of Crete, Greece, the Veterinary Department of the Region of Crete, Greece, under license number 147152 (date 17 July 2017, Heraklion, Crete, Greece). Furthermore, all experiments were in accordance with the International Association for the Study of Hyperalgesia and the paper follows the rules of the Declaration of Helsinki.

### 2.2. Animal Treatment

**Scheme 1:**
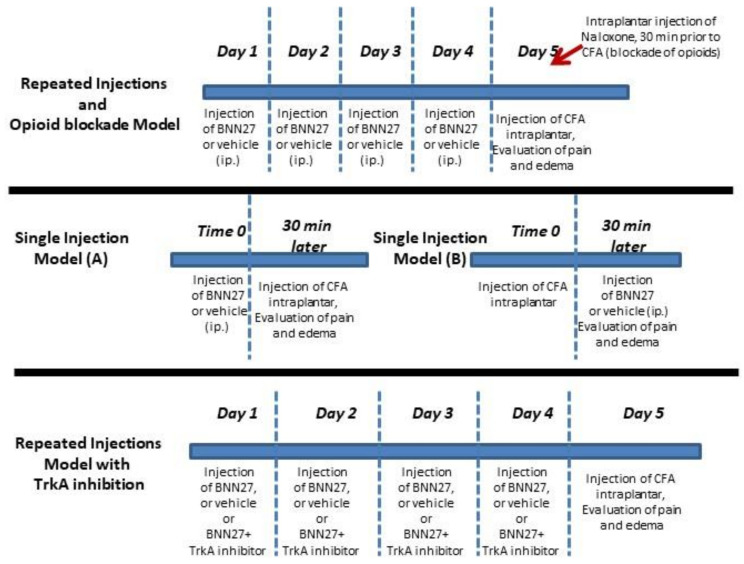
Time and route of BNN27 administration.

To induce inflammatory hyperalgesia, intraplantar injections of 20 μL of CFA (Sigma, Taufkirchen, Germany) into the left hind paw of each mouse were performed [36]. Mice were divided as follows: the BNN27-treated groups which received BNN27 (10 or 100 mg/kg of body weight) intraperitoneally (i.p.) (diluted in 5% DMSO initially, followed by dilution in water for injection at a final volume of 250 μL) and the control group that received injections with the vehicle (5% DMSO and water for injection). BNN27 was manufactured by Calogeropoulou et al. [31] and Bionature EA LTD is the proprietor of the compound. BNN27 was used as received from the company without further purification. The injections with BNN27 or vehicle were performed either repeatedly for four consecutive days, or as a single dose, before (single injection model A) or following the induction of inflammation (single injection model B). CFA was injected 24 h following the last injection of BNN27 in the repeated injections protocol of the compound, while following single administration of BNN27 the interval between BNN27 and CFA injections was 30 min. Similar protocols were followed for the injections with DHEA. To inhibit NGF receptor TrkA, 20 μL of TrkA inhibitor (CAS 388626-12-8) (Merck, Taufkirchen, Germany) (diluted initially in DMSO at 10-2 M, followed by dilution in water for injection at a final concentration 10-5 M) were injected intraplantarly into the left hind paw of mice for four consecutive days simultaneously with BNN27 100 mg/kg (administered i.p.), prior to the induction of the inflammation. The threshold of hyperalgesia was evaluated 24 h post inflammation. Blockade of opioids was achieved by a single intraplantar injection of 20 μL of Naloxone-Hydrochloride (5 mg/kg diluted in water for injection, Research Biochemicals International, Natick, MA) into the left hind paw 30 min before CFA injection [37] in mice treated with BNN27 100 mg/kg for 4 consecutive days. All these procedures are presented in Scheme 1.

### 2.3. Behavioral Testing

Thermal nociceptive thresholds were evaluated using the Hargreaves test [38]: 5 days before the experiment, mice were housed individually and habituated to the plantar environment every day for 30 min/each day (acclimation). Evaluation of inflammatory hyperalgesia thresholds were performed at 3, 6 and 24 h post CFA injections. Inflammatory hyperalgesia is expressed as the time each mouse needs to react to the radiation (IR) that is applied by an IR source beneath the hind paw (paw withdrawal latency). The inflammation-associated edema was measured using a plethysmometer at the same time points. The inflamed paw is immersed in plethysmometer buffer (0.08% NaCl and 0.2% wetting compound) and the volume of the buffer that is displaced is proportional to the size of the edema. All experiments started an hour after the lights had been turned on. 

### 2.4. Measurement of Corticosterone

Following the behavioral experiments, blood was collected from the retroocular vein and centrifuged at 3000 rpm for 10 min at 4 °C. Supernatant was collected and serum corticosterone levels were measured by a RadioImmunoAssay kit (MP Biomedicals, Irvine, CA, USA) according to the instructions of the manufacturer.

### 2.5. Histological Studies 

Mice were euthanized at the time points indicated in the Results section and inflamed hind paws were collected and fixed in 4% paraformaldehyde, followed by immersion in 30% sucrose for cryoprotection. Sections of hind paws were cut (10 μm) in a cryotome and Hematoxylin/Eosin staining was performed. All photographs were taken under 40x magnifications and the scale was 50 μm. All sections were also evaluated by a blind investigator.

### 2.6. Myeloperoxidase (MPO) Assay

Inflamed paws were dissected, weighed and cut in small pieces of the same size. Tissues were homogenized in 300 μL buffer containing 50 mM K_2_PO_4_ pH 6.0 and 0.5% hexadecyltrimethylammonium bromide and were centrifuged at 10,000 rpm at 4 °C for 2 min. In total, 20 μL of the supernatant were mixed with 180 μL of phosphate buffer containing 1 mM of o-dianisidine dihydrochloride and 0.001 % of H_2_O_2_ in a 96-well plate. Samples were incubated in 37 °C for 10 min and the reaction was stopped with 50 μL of H_2_SO_4_. The absorbance was measured in 450 nm taking 3 readings with an interval of 30 s to evaluate the MPO activity [39,40].

### 2.7. Measurement of Cytokines

Inflamed hind paws were homogenized in PBS containing protease inhibitors (Roche, Munich, Gernany). A Bradford assay was performed to determine the total protein concentration of each sample, and quantitation of tissue TNF-α, IL-6, IL-1β and IL-10 levels was carried out using mouse ELISA kits (Biolegend for TNF-α, IL-6, IL-1β; R&D Systems for IL-10) according to the instructions of the manufacturer. For serum cytokine determination, blood was collected as described in the corticosterone evaluation section and ELISA measurements for TNF-α, IL-6 and IL-10 were performed.

### 2.8. Measurement of β-Endorphin

Beta-endorphin was measured with a mouse β-endorphin ELISA kit according to the instructions of the manufacturer (Elabscience, cat# E-EL-MO184). Briefly, inflamed paws were removed, weighed and homogenized in ice-cold phosphate buffer saline, followed by sonication. At first, 50 μL of the standard and samples were added to each well and incubation for 45 min in 37 °C, with 50 μL of Detection Antibody, was performed. The plate was then washed 3 times, and incubated with 100 μL of Conjugated HRP for 30 min in 37 °C. The HRP was washed 5 times and 90 μL of Substrate Reagent was added for 15 min in 37 °C. The reaction was stopped with the specific Stop Solution. The absorbance was determined at 450 nm. Calculation of the concentration of beta-endorphin was carried out by a four-parameter logistic curve. 

### 2.9. Quantitative Real-Time RT-PCR

Total RNA was extracted from inflamed paws with Trizol reagent (Invitrogen, Waltham, MA, USA) and cDNAs were synthesized using a TAKARA PrimeScript 1st strand cDNA synthesis kit (Takara Bio, Saint-Germain-en-Laye, France). Expression of each gene of interest was determined using SYBR Green master mix (Kapa Biosystems, Wilmington, MA, USA) containing specific sets of primers in a final volume of 10 μL. Expression of each gene was normalized to β-actin mRNA. Amplification conditions included denaturation at 95 °C for 2 min followed by 40 cycles at 95 °C for 30 s and at 60 °C for 30 s. The sequence of the primers used is listed in Table 1.

### 2.10. Western Blot

Hind paw extracts were prepared by homogenization in RIPA lysis buffer (0.1% SDS, 1% Igepal CA-630, 1% sodium deoxycholate, 10 mM Tris-HCl, pH 7.5, 150 mM NaCl, 2 μg/mL aprotinin, 1 μg/mL leupeptin, 100 μg/mL PMSF, 0.5 mM EDTA) containing a cocktail of protease and phosphatase inhibitors. Protein concentration was determined by Bradford assay, and 30 μg of protein was loaded for each sample in SDS-page gel 10%. Proteins were transferred to a nitrocellulose membrane and blocked with 5% BSA for 1 h at 4 °C. Following washes in TBS-T, membranes were incubated overnight at 4 °C with antibodies for AKT1 and AKT2 (total/phospho 1:1000, rabbit; Cell Signaling cat# 2964S and 8599S, respectively). To detect the bands of interest, we used the Benchmark Pre-Stained Protein Standard (Invitrogen cat # 10748-010). Normalization was carried out with β-actin (1:5000, mouse; Abcam cat# ab6276). Quantification of gels was performed using the Image-J software. The bands that are presented in the results proceeded from cropping and merging bands from the same original images.

### 2.11. Statistical Analysis

All data were expressed as the mean ± SEM. Experiments were performed independently at least three times and each experiment included at least 3 mice per group (the exact number of mice per experiment is mentioned in the corresponding figure legend). Hyperalgesia thresholds (in seconds) were analyzed using ANOVA followed by post-hoc tests. Unpaired *t*-tests were used in all other comparisons. A *p* value less than 0.05 was assumed to be significant. 

## 3. Results

### 3.1. BNN27 Induced Analgesia in Mice under Inflammatory Conditions

**Figure 2:**
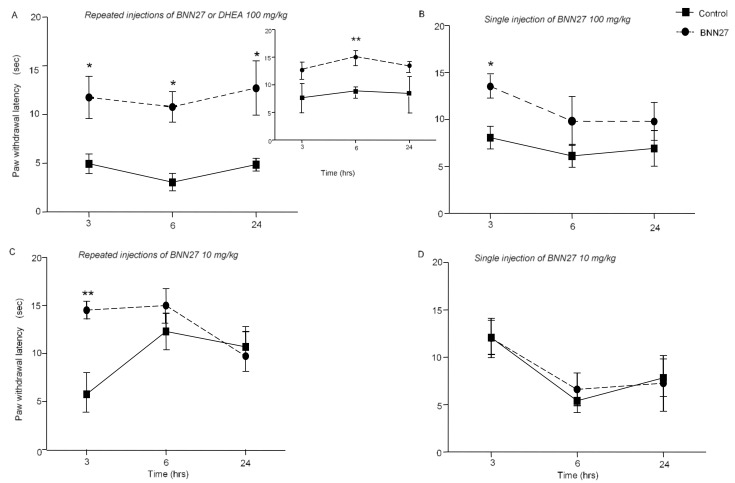
Treatment with BNN27 reduced hyperalgesia in mice at different times and doses examined. (**A**) Repeated injections of 100 mg/kg BNN27 or DHEA (insert graph), following injection of CFA, increased the hyperalgesia threshold. (**B**) Single administration of 100 mg/kg BNN27 was effective 3 h following the induction of the inflammation. (**C**) Repeated injections of 10 mg/kg BNN27 increased paw withdrawal latency, 3 h following the induction of inflammation; however, the same dose was ineffective when administered as a single dose (**D**). (**A**) BNN27 graph: Control n = 5 mice, BNN27-treated group n = 5 mice; DHEA graph: Control n = 4 mice, DHEA-treated group n = 4 mice; (**B**) Control n = 8 mice, BNN27-treated group n = 10 mice; (**C**) Control n = 3 mice, BNN27-treated group n = 4 mice; (**D**) Control n = 3 mice, BNN27-treated group n = 4 mice. In all panels, * *p* < 0.05, ** *p* < 0.01. Statistical analysis was performed using 2-way ANOVA following LSD post-hoc tests. All data are expressed as the mean ± SEM.

We initially confirmed the hyperalgesia induced by the injection of CFA (Supplementary Figure S2A), and then we evaluated the effect of BNN27 at doses 100 and 10 mg/kg of body weight, administered i.p. daily for 4 consecutive days (repeated injections). BNN27 (100 mg/kg) successfully reversed the inflammatory hyperalgesia developed by CFA, as shown by the statistically significant increase in paw withdrawal latency, measured at 3, 6 and 24 h following BNN27 administration (Figure 2A). Repeated injections of 100 mg/kg per body weight of DHEA successfully reversed CFA-induced hyperalgesia in mice at different time points examined (Figure 2A, insert). Repeated injections with 10 mg/kg of ΒΝΝ27 significantly affected paw withdrawal latency only at 3 h following the onset of inflammation (Figure 2C). When BNN27 was administered as a single dose of 100 mg/kg, it was effective only 3 h following the onset of the inflammation (Figure 2B), while a single dose of 10 mg/kg had no effect (Figure 2D). Furthermore, administration of BNN27 (either 100 mg/kg or 10 mg/kg per body weight) as a single dose, 30 min after CFA injections, did not produce any effect (Supplementary File S1B,C).

### 3.2. BNN27 Affected Paw Inflammatory Response 

**Figure 3:**
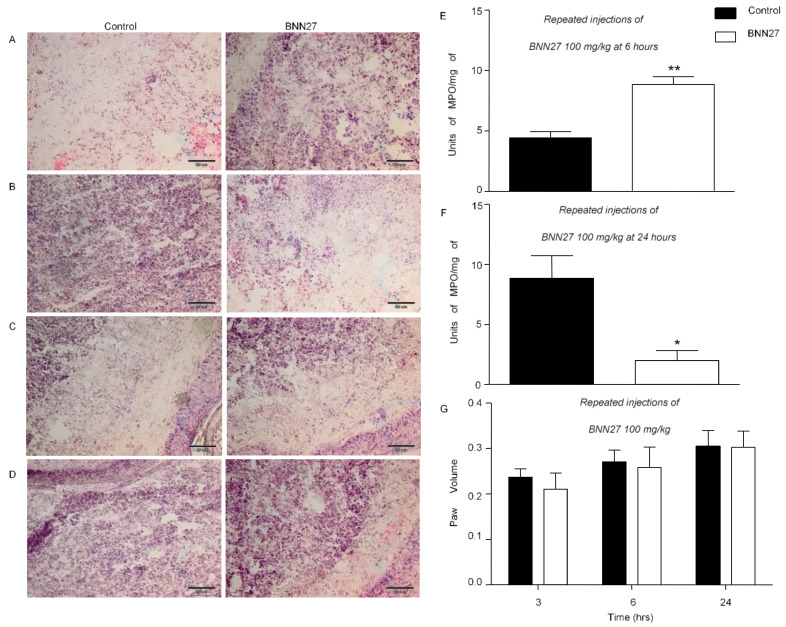
Effect of BNN27 on paw edema and immune cells. Histological examination of the inflamed paw (**A**–**D**). Leukocyte infiltration in the inflamed paw after repeated injections of BNN27 100 mg/kg at 6 (**A**) and 24 (**B**) hours. Repeated injections of BNN27 10 mg/kg at 3 h (**C**). Single administration of BNN27 100 mg/kg at 3 h (**D**). Following repeated injections of BNN27 100 mg/kg MPO activity is increased at 6 h (**E**), while at 24 h the MPO activity decreases in BNN27-treated mice (**F**). Repeated injections of BNN27 at 100 mg/kg did not alter paw edema (**G**). (**A**–**D**) Control n = 5 mice, BNN27-treated group n = 5 mice; (**E**,**F**) Control n = 4 mice, BNN27-treated group n = 4 mice, in both graphs; (**G**) Control n = 9 mice, BNN27-treated group n = 10 mice. In all panels * *p* < 0.05, ** *p* < 0.01. Statistical analysis was performed using unpaired *t*-tests. All data are expressed as the mean ± SEM. Scale bar = 50 μm.

CFA induces not only hyperalgesia, but also paw edema and inflammation [34]. To investigate the effect of BNN27 on paw inflammatory reaction, we assessed leukocyte infiltration in the inflamed paw as well as paw edema. A statistically significant increase in infiltration of immune cells was shown 6 h following the induction of inflammation and treatment with BNN27 (100 mg/kg) for 4 consecutive days (Figure 2A), while treatment with the compound resulted in decreased immune cell infiltration compared to controls at 24 h (Figure 2B). Repeated injections of BNN27 10 mg/kg did not have any effect on leukocyte infiltration at 3 h (Figure 2C). Similarly, a single administration of BNN27 100 mg/kg at 3 h did not alter the leukocytic infiltration (Figure 2D). To confirm our histological data regarding leukocyte infiltration in the inflamed paw of mice injected with BNN27 100 mg/kg for 4 consecutive days, we assessed the MPO activity. We showed that mice treated with repeated injections of BNN27 100 mg/kg increased the MPO activity 6 h following the induction of the inflammation (Figure 3E). In contrast, the MPO activity was decreased in another BNN27-treated group at 24 h post treatment (Figure 2F). However, none of the doses used (single or repeated) had any significant effect on paw edema (Figure 2G). 

### 3.3. BNN27 Affected the Production and Release of Cytokines in the Inflamed Paw and Changed Their Plasma Levels

**Figure 4:**
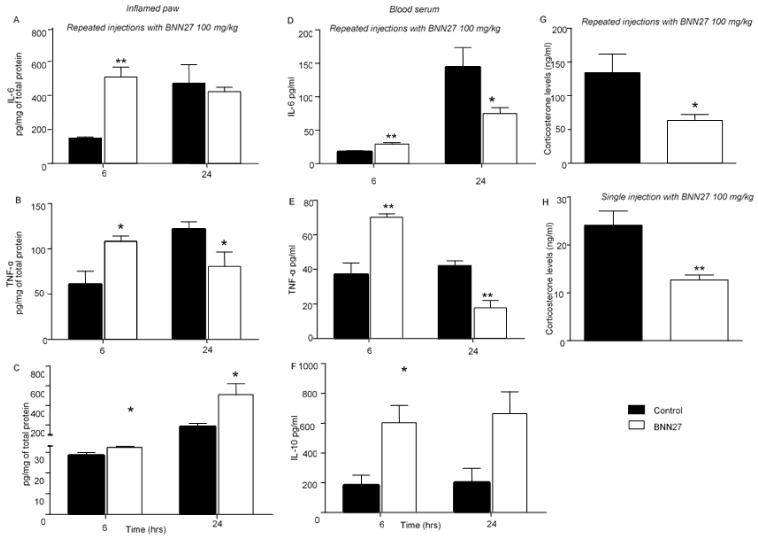
BNN27 affected cytokine production in the inflamed paw and blood serum. Repeated injections of BNN27 100 mg/kg increased the IL-6 and TNF-α levels in the inflamed paw (**A**,**B**,**D**,**E**) at 6 h while it decreased TNF-α at 24 h both in the inflamed paw and blood serum (**B**,**E**). IL-10 remained elevated at all time points examined in the same tissues as above (**C**,**F**). Repeated or single administration of BNN27 100 mg/kg decreased serum levels of corticosterone (**G**,**H**). (**A**) At 6 h: Control n = 3 mice, BNN27-treated group n = 3 mice; at 24 h: Control n = 10 mice, BNN27-treated group n = 10 mice; (**B**) At 6 h: Control n = 4 mice, BNN27-treated group n = 4 mice; at 24 h: Control n = 4 mice, BNN27-treated group n = 5 mice; (**C**) At 6 h: Control n = 4 mice, BNN27-treated group n = 4 mice; at 24 h: Control n = 6 mice, BNN27-treated group n = 5 mice; (**D**) At 6 h: Control n = 4 mice, BNN27-treated group n = 4 mice; at 24 h: Control n = 3 mice, BNN27-treated group n = 4 mice; (**E**) At 6 h: Control n = 3 mice, BNN27-treated group n = 4 mice; at 24 h: Control n = 3 mice, BNN27-treated group n = 3 mice; (**F**) At 6 h: Control n = 4 mice, BNN27-treated group n = 4 mice; at 24 h: Control n = 3 mice, BNN27-treated group n = 4 mice; (**G**) Control n = 9 mice, BNN27-treated group n = 8 mice; (**H**) Control n = 3 mice, BNN27-treated group n = 4 mice. In all panels * *p* < 0.05, ** *p* < 0.01. Statistical analysis was performed using unpaired *t*-tests. All data are expressed as the mean ± SEM.

Tissue (inflamed paw) and plasma cytokine levels were evaluated 6 and 24 h following treatment with BNN27. Repeated injections of BNN27 at the concentration of 100 mg/kg resulted in significantly elevated release of pro-inflammatory cytokines (IL-6 and TNF-α) and the anti-inflammatory cytokine IL-10 at 6 h following the induction of the inflammation (Figure 4A–C). At 24 h after the induction of inflammation, paw tissue TNF-α levels were significantly lower in the BNN27-treated mice compared to the vehicle-treated mice (Figure 4B) while the IL-6 tissue levels did not differ between the two groups (Figure 4A). Finally, the IL-10 levels remained elevated in BNN27-treated (Figure 4C) compared to vehicle-treated mice. In plasma, increased levels of IL-6 (Figure 4D), TNF-α (Figure 4E) and IL-10 (Figure 4F) were observed at 6 h in the BNN27-treated in comparison with the vehicle-treated mice. At 24 h, plasma levels of IL-6 and TNF-α were significantly reduced in mice treated with BNN27 (Figure 4D,E). Furthermore, the IL-10 levels were found elevated (Figure 4F). Evaluation of IL-1β in the inflamed paw following repeated injections of BNN27 100 mg/kg did not show any significant differences between groups at 24 h (Supplementary File S1D). To examine whether the effect of BNN27 on cytokine levels was mediated through regulation of endogenous glucocorticoids, we measured in parallel the plasma levels of corticosterone. No significant differences were found in the plasma corticosterone levels at 6 h following the induction of inflammation between the vehicle- and BNN27-treated mice (Supplementary File S1E). However, at 24 h, the plasma corticosterone levels were significantly lower in mice that were treated with repeated injections of BNN27 100 mg/kg compared to the vehicle-treated mice (Figure 4G). Significantly decreased plasma corticosterone levels were also detected in the BNN27-treated mice following injection of a single dose of 100 mg/kg (Figure 4H). 

We also assessed the effect of repeated injections of BNN27, at a dose of 10 mg/kg, on both the paw and plasma cytokine levels. This dose resulted in significantly decreased inflamed paw TNF-α levels compared to the vehicle-treated mice but had no effect on the IL-6 and IL-10 levels, both in inflamed paws and plasma (Supplementary File S2). Finally, single administration of BNN27 at a dose of 100 mg/kg did not have any effect on the protein levels of these cytokines, both in the inflamed paw and plasma (Supplementary File S3).

### 3.4. BNN27 Affects the Production of NGF and iNOS in the Inflamed Paw

**Figure 5:**
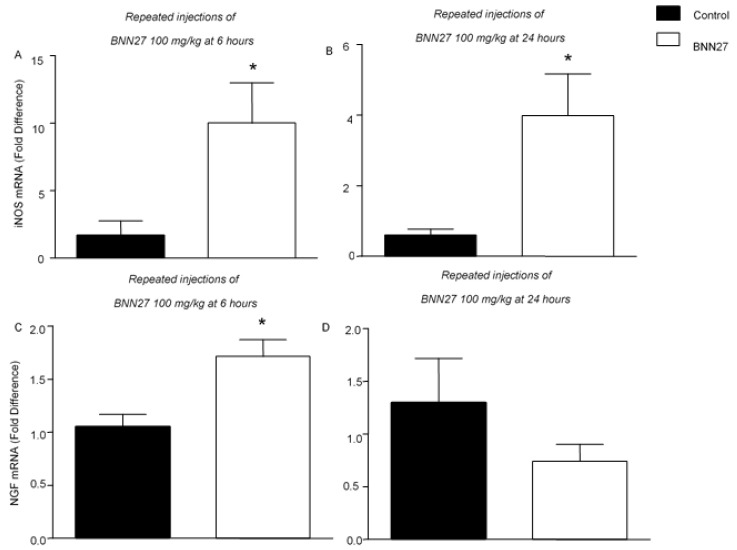
Effect of BNN27 on inflammatory mediators. Repeated injections with BNN27 100 mg/kg increased the iNOS mRNA levels in the inflamed paw at 6 (**A**) and 24 (**B**) hours following CFA injection. NGF mRNA in the inflamed paw was increased following treatment with BNN27 100 mg/kg at 6 h (**C**) but not at 24 (**D**). (**A**) Control n = 4 mice, BNN27-treated group n = 4 mice; (**B**) Control n = 4 mice, BNN27-treated group n = 5 mice; (**C**) Control n = 4 mice, BNN27-treated group n = 4 mice; (**D**) Control n = 4 mice, BNN27-treated group n = 5 mice. In all panels * *p* < 0.05. Statistical analysis was performed using unpaired *t*-tests. All data are expressed as the mean ± SEM.

NGF and nitric oxide (NO) are key factors of inflammation and hyperalgesia [41]. We therefore evaluated the mRNA levels of NGF and iNOS in the inflamed paw following repeated injections with 100 mg/kg of BNN27. As shown in Figure 5, BNN27 treatment significantly increased the iNOS and NGF mRNA levels compared to the vehicle-treated mice 6 h following the onset of inflammation (Figure 5A,C). At 24 h, the iNOS mRNA levels remained elevated in the BNN27-treated mice (Figure 5B); however, no significant changes in the NGF mRNA levels were observed between groups at this time point (Figure 5D).

### 3.5. BNN27 Affects AKT Signaling Pathway

**Figure 6:**
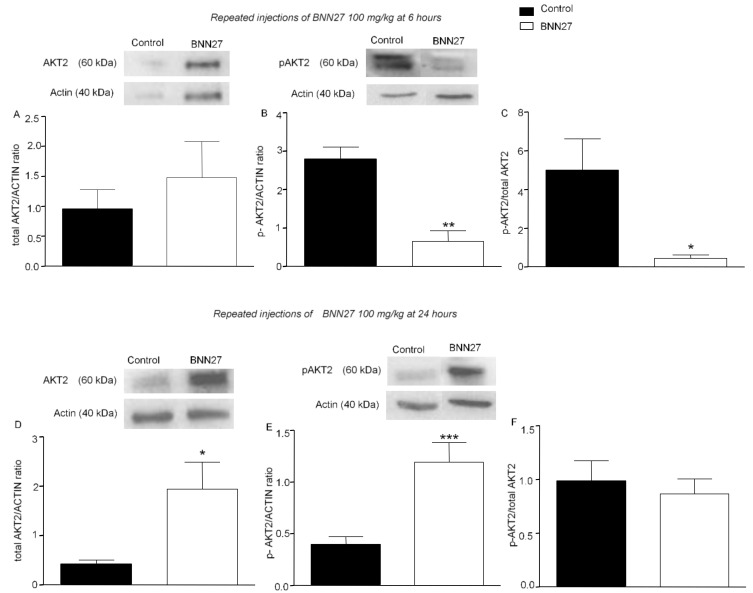
BNN27 affected AKT2 phosphorylation in the inflamed paw. Repeated injections of BNN27 100 mg/kg decreased AKT2 phosphorylation at 6 h but had no effect on total AKT2 (**A**–**C**). At 24 h, BNN27 increased the expression of total (**D**) and p-AKT2 (**E**) but the overall AKT2 phosphorylation was unchanged (**F**). (**A**) Control group n = 4 mice, BNN27 treated group n = 4 mice; (**B**) Control group n = 3 mice, BNN27-treated group n = 3 mice; (**C**) Control group n = 3 mice, BNN27-treated group n = 3 mice; (**D**) Control group n = 4 mice, BNN27-treated group n = 4 mice; (**E**) Control group n = 4 mice, BNN27-treated group n = 3 mice; (**F**) Control group n = 3 mice, BNN27-treated group n = 3 mice. In all panels * *p* < 0.05, ** *p* < 0.01, *** *p* < 0.001. Statistical analysis was performed using unpaired *t*-tests. All data are expressed as the mean ± SEM.

Previous studies showed that BNN27 influences the TrkA receptor downstream signaling pathway of AKT kinase [30]. In our study, we evaluated the effect of BNN27 on the levels of total and phosphorylated AKT1 and AKT2 proteins. Administration of BNN27 100 mg/kg for four consecutive days did not significantly alter the total AKT2 levels (Figure 6A), but reduced the phosphorylated AKT2 levels (Figure 6B) and the ratio of the phosphorylated form of AKT2 to total AKT2 (Figure 6C), 6 h after the induction of inflammation. At 24 h, the expression of total and phosphorylated AKT2 in the BNN27-treated mice was significantly increased, but the overall levels of the phosphorylated form of AKT2 did not have any significant alterations (Figure 6D–F). No significant changes were observed in total or phosphorylated levels of AKT1 proteins at any time point tested (Supplementary File S4). 

### 3.6. Effect of BNN27 on the Opioid System

**Figure 7:**
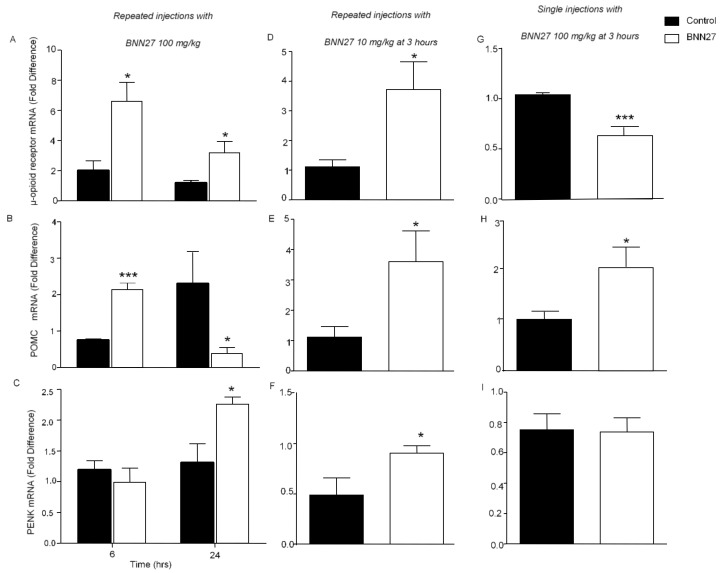
BNN27 induced the expression of opioid peptides and μ-opioid receptor in the inflamed paw. Repeated injections of 100 mg/kg increased the μ-opioid receptor mRNA levels at 6 and 24 h (**A**) as well as POMC mRNA levels at 6 h (**B**) and PENK mRNA levels at 24 h (**C**). Repeated injections of 10 mg/kg increased μ-opioid receptor, POMC and PENK mRNA levels at 3 h (**D**–**F**). Single treatment at 100 mg/kg decreased the μ-opioid receptor mRNA (**G**) and increased the POMC mRNA levels (**H**). No changes were observed in the PENK mRNA levels (**I**). (**A**) At 6 h, Control n = 3 mice, BNN27-treated group n = 4 mice; at 24 h: Control n = 4 mice, BNN27-treated group n = 4 mice. (**B**) At 6 h, POMC: Control n = 3 mice, BNN27-treated group n = 5 mice; at 24 h: Control n = 5 mice, BNN27-treated group n = 5 mice. (**C**) At 6 h, Control n = 4 mice, BNN27-treated group n = 4 mice; at 24 h: Control group n = 4 mice, BNN27-treated group n = 4 mice. (**D**) Control n = 5 mice, BNN27-treated group n = 5 mice. (**E**) Control n = 5 mice, BNN27-treated group n = 5 mice. (**F**) Control n = 4 mice, BNN27-treated group n = 5 mice. (**G**) Control n = 4 mice, BNN27-treated group n = 5 mice. (**H**) Control n = 4 mice, BNN27-treated group n = 5 mice. (**I**) Control n = 4 mice, BNN27-treated group n = 5 mice. In all panels * *p* < 0.05, *** *p* < 0.001. Statistical analysis was performed using unpaired *t*-tests. All data are expressed as the mean ± SEM.

To define the possible involvement of the opioid system in the analgesic effects of BNN27, we measured the levels of the mRNAs of opioid peptides and receptors in the inflamed paw, using quantitative PCR analysis. Our data showed that treatment with BNN27 (100 mg/kg) for 4 consecutive days significantly increased the mRNA levels of the μ-opioid receptor and proopiomelanocortin (POMC) at 6 h and the levels of proenkephalin (PENK) at 24 h (Figure 7A–C). Surprisingly, the levels of the POMC mRNA at 24 h were found significantly reduced (Figure 7B). Administration of BNN27 at 10 mg/kg for four consecutive days resulted in elevated levels of the mRNAs of the μ-opioid receptor, PENK and POMC at 3 h (Figure 7D–F), whereas single treatment with 100 mg/kg resulted in increased POMC mRNA levels at 3 h (Figure 7H). 

**Figure 8:**
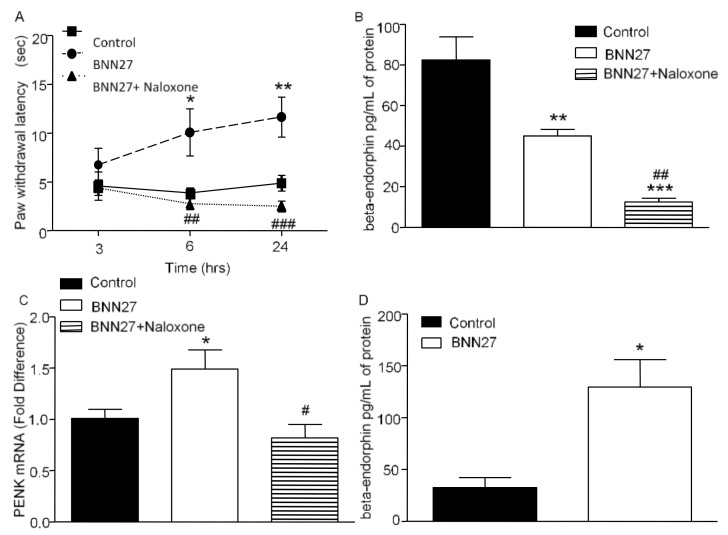
Antagonism of opioids with naloxone reversed the analgesic effect of BNN27. Administration of naloxone following treatment with repeated injections of BNN27 100 mg/kg diminished paw withdrawal latency (**A**). Beta- endorphin and PENK was found decreased in naloxone-treated mice compared to mice treated only with BNN27 100 mg/kg at 24 h ((**B**,**C**), respectively). At 6 h, administration of BNN27 100 mg/kg for 4 days increased paw beta-endorphin (**D**). (**A**–**C**) Control n = 4 mice, BNN27-treated group n = 4 mice, BNN27+Naloxone-treated group n = 6 mice; (**D**) Control n = 4 mice, BNN27-treated group n = 4 mice. In all panels * *p* < 0.05, ** *p* < 0.01, *** *p* < 0.001, # *p* < 0.05, ## *p* < 0.01 and ### *p* < 0.001. In panels (**A**–**C**), an asterisk (*) depicts a comparison between the Control and BNN27-treated group, and # depicts a comparison between the BNN27-treated group and BNN27+Naloxone-treated group. Statistical analysis in panel A was performed using 2-way ANOVA followed by an LSD post-hoc test. Statistical analysis for panels (**B**,**C**) was performed using 1-way ANOVA followed by a Newman–Keuls post-hoc test. Statistical analysis for panel (**D**) was performed using an unpaired *t*-test. All data are expressed as the mean ± SEM.

To confirm the involvement of the opioid system in the analgesic effect of BNN27, we administered a single intraplantar injection of the opioid antagonist naloxone, 30 min prior the CFA injection, in mice which were treated repeatedly with 100 mg/kg of BNN27. Our data showed that naloxone injection resulted in dramatically reduced paw withdrawal latency in mice treated with BNN27 compared to those treated with BNN27 only (Figure 8A). In addition, we measured the paw β-endorphin protein levels and PENK mRNA levels following naloxone treatment. Co-administration of BNN27 and naloxone resulted in significantly reduced β-endorphin protein levels and PENK mRNA levels compared to the BNN27-only treated mice 24 h from the onset of inflammation (Figure 8B,C respectively). Since the POMC mRNA levels were found elevated at 6 h following repeated injections of BNN27 100 mg/kg, we also evaluated β-endorphin at this time point as well. Our results demonstrated that the BNN27-treated mice exhibited increased serum β-endorphin levels compared to the controls (Figure 8D) following CFA injections.

### 3.7. Effect of BNN27 on NGF and Its Receptors in Dorsal Root Ganglia (DRG)

**Figure 9:**
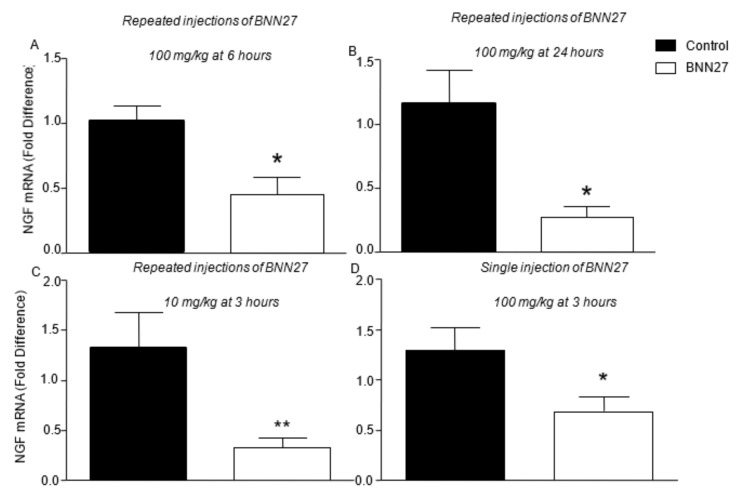
BNN27 inhibited the expression of NGF in DRG at all time points and doses tested. (**A**) Repeated injections of BNN27 at 100 mg/kg at 6 h: Control n = 3 mice, BNN27-treated group n = 3 mice. (**B**) Repeated injections of BNN27 100 mg/kg at 24 h: Control n = 6 mice, BNN27-treated group n = 5 mice. (**C**) Chronic treatment with BNN27 10 mg/kg: Control n = 8 mice, BNN27-treated group n = 10 mice. (**D**) Single treatment with BNN27 100 mg/kg: Control n = 7 mice, BNN27-treated group n = 9 mice In all panels * *p* < 0.05, ** *p* < 0.01. Statistical analysis was performed using unpaired *t*-tests. All data are expressed as the mean ± SEM.

It is well documented that CFA triggers the production of NGF not only at the site of inflammation, but also in DRG, enhancing the hyperalgesia signals [42]. In our study, the mRNA levels of NGF were found significantly decreased, both following repeated injections of BNN27, either at 100 or 10 mg/kg (Figure 9A–C), and single treatment with BNN27 at 100 mg/kg (Figure 9D) in DRG. 

**Figure 10:**
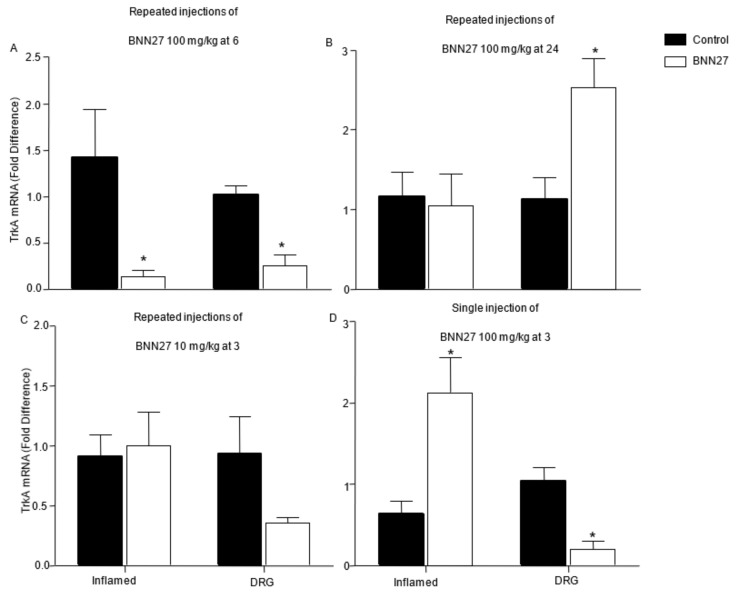
Effect of BNN27 on the expression of TrKA in the inflamed paw and DRG. Repeated injections of 100 mg/kg of BNN27 decreased the TrkA mRNA levels in the inflamed paw and DRG at 6 h (**A**), while at 24 h BNN27 had no effect on paw TrkA mRNA whereas it increased its expression in DRG (**B**). Repeated injections of 10 mg/kg of BNN27 did not induce any significant changes in the TrkA mRNA levels in the inflamed paw and DRG (**C**). Single treatment with 100 mg/kg of BNN27 increased paw TrkA mRNA levels while in DRG, the same dose decreased TrkA mRNA levels (**D**). (**A**) Inflamed paw: Control n = 4 mice, BNN27-treated group n = 4 mice; DRG: Control n = 4 mice, BNN27-treated group n = 4 mice. (**B**) Inflamed paw: Control n = 4 mice, BNN27-treated group n = 5 mice; DRG: Control n = 5 mice, BNN27-treated group n = 4 mice. (**C**) Inflamed paw: Control n = 6 mice, BNN27-treated group n = 5 mice; DRG: Control n = 6 mice, BNN27-treated group n = 5 mice. (**D**) Inflamed paw: Control n = 4 mice, BNN27-treated group n = 4 mice; DRG: Control n = 4 mice, BNN27-treated group n = 4 mice. In all figures * *p* < 0.05. Statistical analysis was performed using unpaired *t*-tests. All data are expressed as the mean ± SEM.

The mRNA levels of the NGF receptor, TrkA, were also evaluated in the inflamed paw and DRG, after administration of BNN27. Significantly decreased mRNA levels of TrkA were found in the inflamed paw and DRG of mice treated with BNN27 100 mg/kg for four consecutive days 6 h after the induction of the inflammation (Figure 10A), but at 24 h the mRNA levels of TrkA were significantly increased only in DRG of the BNN27-treated mice compared to the controls, while no significant changes observed in the mRNA levels of TrkA in the inflamed paw at that concentration of BNN27 used (Figure 10B). Repeated injections of BNN27 at 10 mg/kg did not significantly affect the mRNA levels of TrkA, either in the inflamed paw or the DRG at three hours (Figure 10C). Administration of a single dose of BNN27 at 100 mg/kg resulted in a significant increase of TrkA mRNA in paws, but in DRG its levels were found significantly reduced, compared to the controls (Figure 10D). BNN27 did not alter the levels of the mRNA of the low affinity receptor of NGF, p75 NTR (Supplementary File S5A,B) or substance P (Supplementary File S5C,D), at any condition tested and tissue tested.

### 3.8. Effect of BNN27 Following Inhibition of TrkA

**Figure 11:**
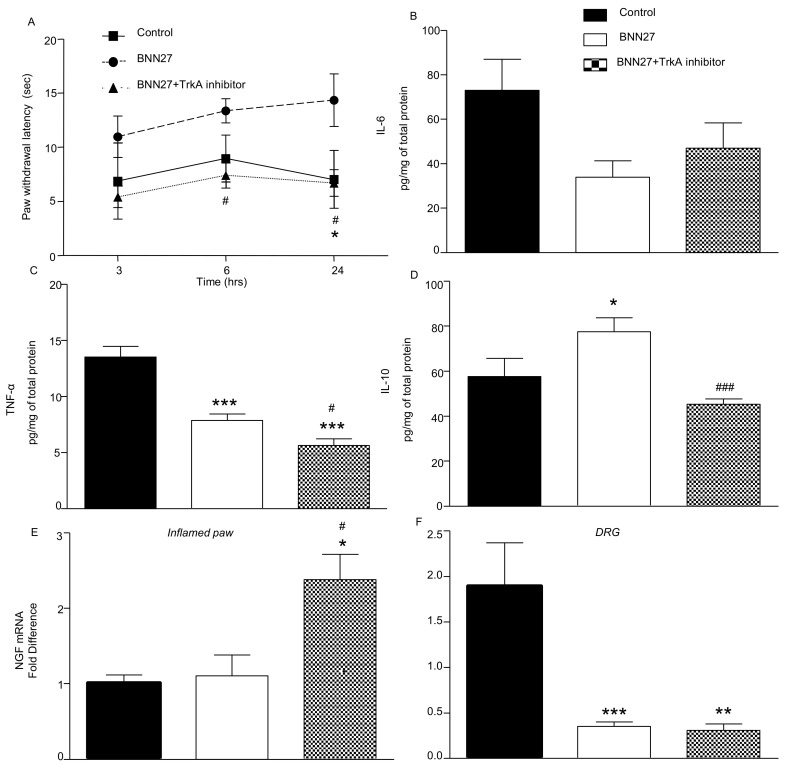
Inhibition of TrkA decreased the pain threshold in BNN27-treated mice and affected the inflammatory mediators in the inflamed paw. TrkA inhibitor decreased the effect of BNN27 on paw withdrawal (**A**) and TNF-a and IL-10 protein in the inflamed paw (**C**,**D**), but had no effect on IL-6 (**B**). Paw NGF mRNA levels were found elevated following inhibition of TrkA (**E**), while in DRG its levels were remained significantly decreased (**F**). (**A**–**E**) Control n = 3, BNN27-treated group n = 4, BNN27+TrkA inhibitor-treated group n = 7; (**F**) Control n = 3, BNN27-treated group n = 4, BNN27+TrkA inhibitor-treated group n = 5. An asterisk (*) depicts a comparison between the Control and BNN27-treated group, and # depicts a comparison between the BNN27-treated group and BNN27+TrkA inhibitor-treated group. In all panels * *p* < 0.05, ** *p* < 0.01, *** *p* < 0.001, # *p* < *0.05* and ### *p* < 0.001. Statistical analysis in panel A was performed using 2-way ANOVA followed by an LSD post-hoc test. Statistical analysis for panels B–F was performed using 1-way ANOVA followed by a Newman–Keuls post-hoc test. All data are expressed as the mean ± SEM.

TrkA receptors have been shown to mediate most of the effects of BNN27 in several systems [32,33]. We thus tested the role of TrkA in the analgesic effect of BNN27 in our system. Repeated injections of BNN27 100 mg/kg and the TrkA inhibitor, simultaneously, did not significantly alter the paw withdrawal latency of the treated mice compared to the controls (Figure 11A) at any time point tested. It is of interest that co-administration of the TrKA inhibitor with BNN27 significantly decreased the levels of TNF-α and IL-10 24 h from the induction of the inflammation (Figure 11C,D, respectively), while the levels of IL-6 remained unchanged (Figure 11B). Furthermore, the mRNA levels of NGF in the inflamed paw were found significantly elevated in mice co-receiving the TrkA inhibitor (Figure 11E), whereas in DRG the NGF mRNA levels remained significantly lower following inhibition of TrkA (Figure 11F). It is of note that no significant changes were observed in the levels of the mRNAs of opioid peptide precursors and receptors after co-administration of BNN27 with the TrkA inhibitor (Supplementary File S6). 

## 4. Discussion

In the present study, we evaluated the role of the new synthetic neurosteroid BNN27 on inflammatory hyperalgesia. Our results provide evidence for the first time that BNN27 alleviates from hyperalgesia in mice under inflammatory conditions, an effect which appears to be unrelated to glucocorticoids. We also found that BNN27 controls the inflammatory response although it does not affect the edema, and triggers opioids synthesis and release at the site of inflammation. The effect of BNN27 is likely mediated by the NGF receptor TrkA and the activation of the AKT2 signaling pathway.

Accumulating evidence suggests that neurosteroids are implicated in the process of pain. For instance, pregnenolone (PREG) and DHEA are released in the spinal cord and prevent thermal and mechanical hyperalgesia during carrageenan-induced inflammation in rats [22]. Similar results have been observed with allopregnanolone, which has been shown to provide relief from thermal hyperalgesia evoked from diabetes in rats [43]. It is of note that intraperitoneal administration of DHEA increases the pain threshold in mice and rats [44]. The chronic analgesic effect of DHEA in that study was attributed to androgenic metabolites generated by the daily administration of DHEA, inducing a rapid pro-nociceptive action, while intrathecal administration of testosterone, an androgen deriving from DHEA, caused analgesia in neuropathic rats [45]. Based on the limitations of the clinical use of DHEA because of its multiple secondary effects via its binding to steroid receptors (estrogen receptors alpha and beta, ARs) [46] and its role as a precursor in the biosynthesis of androgens and estrogens [46], we used in our studies BNN27, a new synthetic DHEA analogue. BNN27 is not metabolized to androgens or estrogens, does not interact with steroid receptors [31] and binds specifically to TrkA and p75NTR receptors [30,31]. Our data demonstrated that BNN27 holds analgesic effects when it is used in the model of CFA-induced hyperalgesia, either in repeated doses model or as a single dose.

During the CFA-induced inflammation model, pro-inflammatory cytokines, such as IL-6 and TNF-α, are produced [2,47], enhancing the pain response and sensitizing the nociceptors [8]. Furthermore, central administration of IL-6 or TNF-α in rats induces thermal hyperalgesia [7], and injection of TNF-α or IL-6 in the hind paw of rats evokes hyperalgesia, mimicking the actions of carrageenan [48]. Our data point towards a biphasic effect of BNN27 during inflammatory hyperalgesia which could be attributed to the different receptors mediating the effect at each time point. Indeed, its repeated administration at the dose of 100 mg/kg was accompanied by a remarkable immune response at 6 h, as depicted by the significantly elevated levels of pro-inflammatory cytokines (IL-6, TNF-α, Figure 4) along with the anti-inflammatory cytokine IL-10, not only at the site of inflammation but also in systemic circulation. Similarly, levels of other inflammatory mediators, such as NGF and iNOS, increased in the inflamed paw (Figure 5). However, this increase subsided 24 h following the induction of the inflammation. Additionally, repeated administration of BNN27 augmented the accumulation of leukocytes at the site of the inflammation, 6 h following its onset, which was reversed at 24 h (Figure 3). At that time point, we noticed that the BNN27-treated mice exhibited less infiltration of immune cells compared to the controls, an observation further confirmed by the assessment of MPO activity. 

The anti-inflammatory effects of neurosteroids have been extensively examined. Indeed, DHEA was shown to exert anti-inflammatory effects in the LPS-induced inflammation model, by decreasing the serum TNF-α [49]. In accordance with our findings, administration of DHEA and DHEA sulfate was shown to effectively increase the serum levels of IL-10 in mice [50]. Interestingly, a recent study has shown that BNN27 efficiently reduced TNF-α in the inflamed-retina of diabetic rats, while it increased the anti-inflammatory IL-10 within the same tissue [32]. Several studies have suggested that there is a negative interaction between IL-6 and DHEA, leading to low levels of IL-6 [25,51,52]. It appears that its synthetic analog BNN27 maintains these very interesting anti-inflammatory properties, effectively controlling the levels of IL-6. Indeed, BNN27 initially increased the IL-6 protein levels 6 h following the onset of the inflammation in the inflamed paw and blood serum; however, at 24 h, BNN27 effectively reduced the IL-6 levels in treated mice. It is of interest that the effect of BNN27 is independent of glucocorticoids, since the corticosterone levels were significantly decreased following treatment with the compound contrary to DHEA, which seems to increase the serum levels of corticosterone in a model of stress-induced analgesia in rats [53]. 

Previous studies have shown that during inflammation immune cells produce opioid peptides, which compromise the pain response, while the mRNA of the opioid peptides POMC, PENK and the μ-receptor were also detected in the inflamed paw [54,55,56,57,58]. Our study shows that administration of BNN27, either repeatedly or as a single dose, significantly increased the mRNA levels of all three components of the opioid system as well as β-endorphin in the inflamed paw (Figure 7). The analgesic effect of BNN27 is further supported by effective reversal of its effect by the opioid antagonist naloxone, accompanied by significantly reduced levels of β-endorphin and PENK in the inflamed paw (Figure 8). These findings provide, for the first time, clear evidence on the involvement of the opioid system in the analgesic effects of endogenous and synthetic neurosteroids.

NGF has been characterized as a very important hyperalgesic factor, exerting hyperalgesia in rodents’ paws, too [42,59]. In our study, we described elevated mRNA levels of NGF in the paw of mice, 6 h after treatment with BNN27 (Figure 5). Interestingly, elevated NGF expression was reported in DRG, facilitating the transmission of pain signals [39,54]. The mechanisms of analgesic actions of BNN27 seem to involve the expression in DRG of NGF and its TrkA receptor. Indeed, our findings support this hypothesis, since BNN27 significantly decreased the NGF mRNA levels in DRG at all conditions and doses examined, thus preventing pain signals to be transmitted and induce hyperalgesia (Figure 9). In previous studies, BNN27 was shown to bind and activate solely the TrkA receptor and not TrkB or TrkC receptors [30]. Definitely, inhibition of TrkA in the inflamed paw diminished the analgesic and anti-inflammatory effect of BNN27 in mice treated with BNN27 and the TrkA inhibitor simultaneously (Figure 11). Additionally, the protein levels of the anti-inflammatory cytokine IL-10 declined and these of the NGF mRNA increased in the inflamed paw of mice treated with BNN27 in the presence of the TrkA inhibitor. 

DHEA was shown to reduce microglia activation and inflammation through activation of the Akt signaling pathway involving Akt1/Akt2 phosphorylation [25]. BNN27 was found to reduce the phosphorylation of AKT2 but not AKT1 kinase in the inflamed paw (Figure 6), suggesting a regulatory effect on this downstream to TrkA receptors pathway. These findings further support the anti-inflammatory analgesic effect of BNN27. Indeed, activation of the AKT2 signaling pathway strongly enhances inflammation, while AKT2 deficiency contributes to injury, fibrosis and inflammation [60]. 

## 5. Conclusions

Our findings suggest that the synthetic DHEA analogue, BNN27, exhibits strong analgesic and anti-inflammatory effects, mediated by the activation of the opioid system and the inhibition of NGF in DRG. Its mechanism of action involves the activation of TrkA receptors and downstream AKT2 signaling. Inflammatory pain is the major cause of suffering in various pathophysiological conditions, such as arthritis, infections, cancer, trauma, etc. In the first line of treatment are non-steroidal anti-inflammatory drugs (NSAID), glucocorticoid and opioids; however, their prolonged use has been shown to result in significant gastrointestinal side effects, electrolyte and metabolic disturbances and addiction [61,62]. Our findings suggest that BNN27 is a promising lead molecule to develop new therapies for the relief of pain and the control of inflammation. 

## Figures and Tables

**Table 1:** Sequences of the RT-PCR primers.

Gene	Forward	Reverse
*beta-actin*	tctctttgatgtcacgcacg	tcagaaggactcctatgtgg
*Pomc*	gctgcttcagacctccatagatgtg	cagcgagaggtcgagtttgc
*Penk*	cgacatcaatttcctggcgt	agatccttgcaggtctccca
*μ-opioid receptor*	acgctcagacgttccattct	tccaaagaggcccactacac
*Ngf*	cacccacccagtcttcc	ctcggcacttggtctcaaa
*Trka*	cctgcaacgcttggagtttg	cactcttcacgatggttaggct
*iNOS*	tcctggaggaagtgggccaag	cctccacgggcccggtactc

## Data Availability

The datasets used and/or analyzed during the current study are available from the corresponding author on reasonable request.

## Supplementary Materials

The following are available online at https://www.mdpi.com/article/10.3390/biomedicines9091185/s1, Figure S1: Effect of different doses of BNN27 on paw withdrawal latencies, IL-1β and corticosterone levels (6hrs), Figure S2: Effect of repeated injections of BNN27 10 mg/Kg on cytokine levels in the inflamed paw and blood at 3 h post CFA injections, Figure S3: Effect of a single injection of BNN27 100 mg/kg on cytokine levels in the inflamed paw and blood at 3 h post CFA injections, Figure S4: Repeated injections of BNN27 100 mg/kg did not affect AKT1 signaling pathway, Figure S5: mRNA levels of TrkA receptor p75 and substance p remained unaffected by BNN27 treatment, Figure S6: Administration of BNN27 100 mg/kg simultaneously with TrkA inhibitor did not affect μ-opioid receptor and PENK mRNA compared to mice treated only with BNN27.
