# Supplementary material for: Analgesic and Anti-Inflammatory Effects of the Synthetic Neurosteroid Analogue BNN27 during CFA-Induced Hyperalgesia
Source: Biomedicines. 2021 Sep 9;9(9):1185. doi: 10.3390/biomedicines9091185 (PMC8469064; doi:10.3390/biomedicines9091185)
Supplement: Supplementary file 1 [file biomedicines-09-01185-s001.zip › biomedicines-1342649-supplementary.pdf]

CFA-injected paws exhibit decreased paw withdrawal latency compared to non-injected paws

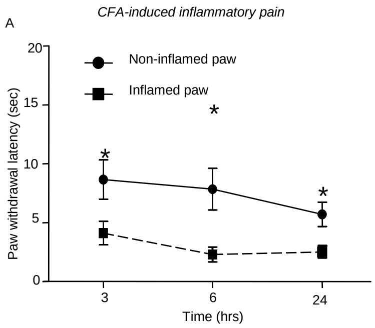

Repeated injections of BNN27 100 mg/kg after the induction of inflammation did not have any effect on paw withdrawal latency

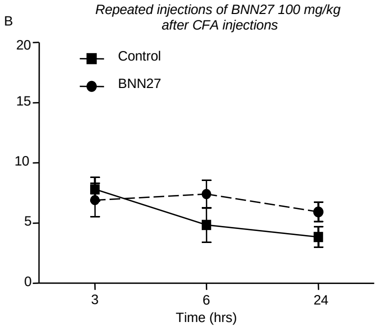

Single injection of BNN27 100 mg/kg after the induction of inflammation did not have any effect on paw withdrawal latency

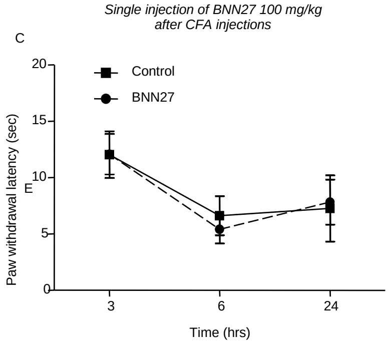

BNN27 administration did not change IL-1β protein levels at any time point and route of administration examined

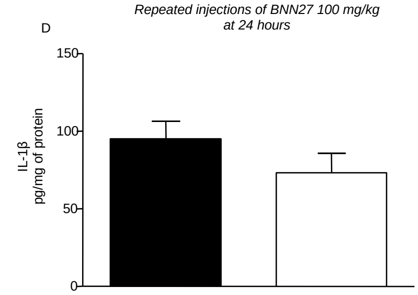

Serum corticosterone levels remained unchanged following repeated injections of BNN27 100 mg/kg at 6 hours

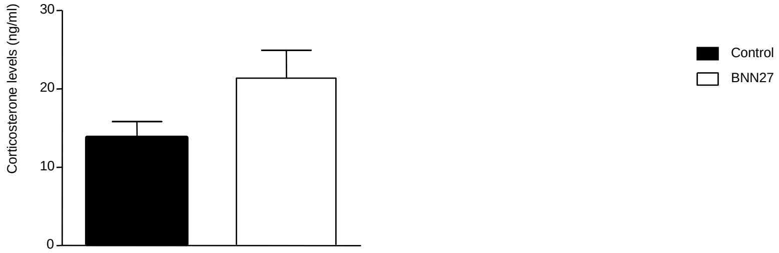

Figure S1: Effect of different doses of BNN27 on paw withdrawal latencies, IL-1β and corticosterone levels (6hrs).

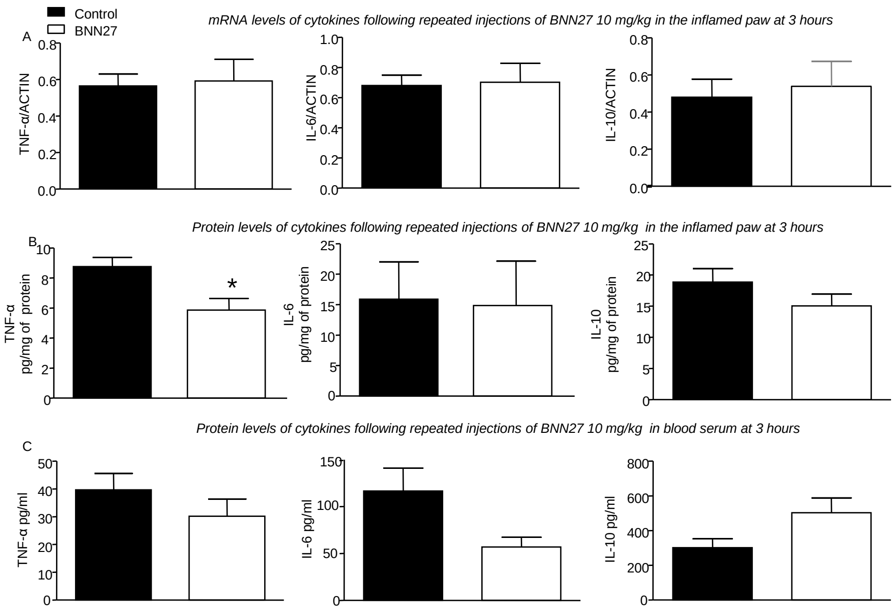

Figure S2: Effect of repeated injections of BNN27 10 mg/Kg on cytokine levels in the inflamed paw and blood at 3 hours post CFA injections.

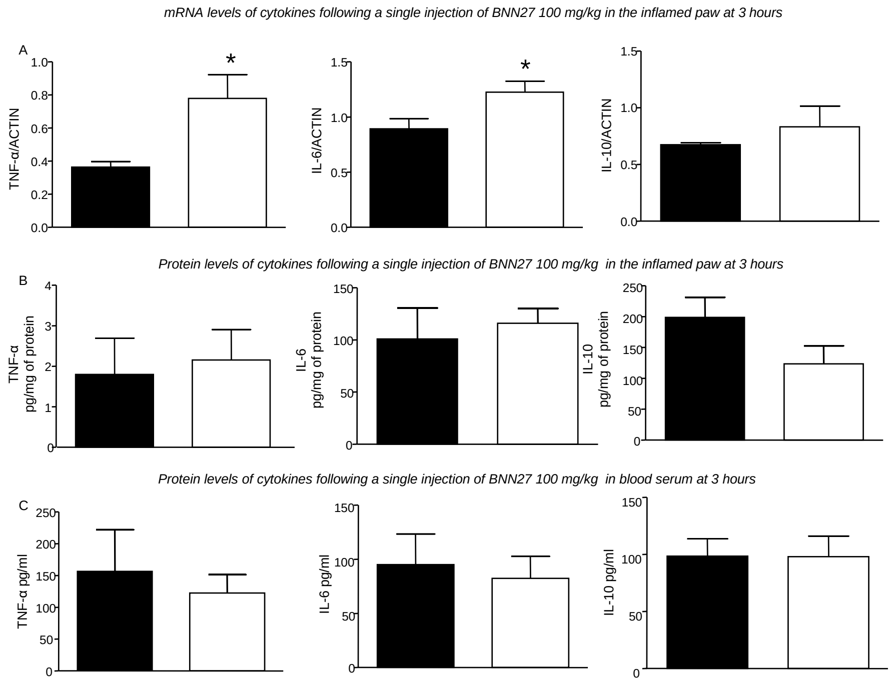

Figure S3: Effect of a single injection of BNN27 100 mg/kg on cytokine levels in the inflamed paw and blood at 3 hours post CFA injections

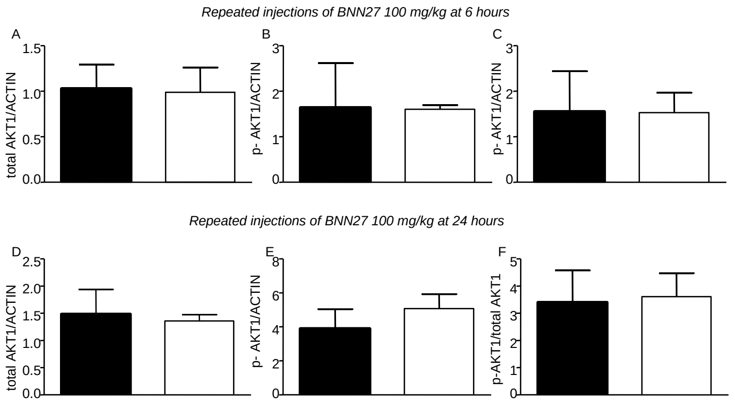

Figure S4: Repeated injections of BNN27 100 mg/kg did not affect AKT1 signaling pathway

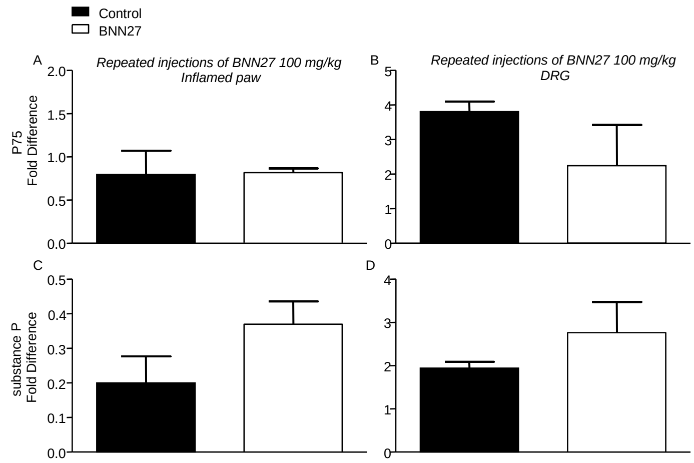

Figure S5: mRNA levels of TrkA receptor p75 and substance p remained unaffected by BNN27 treatment

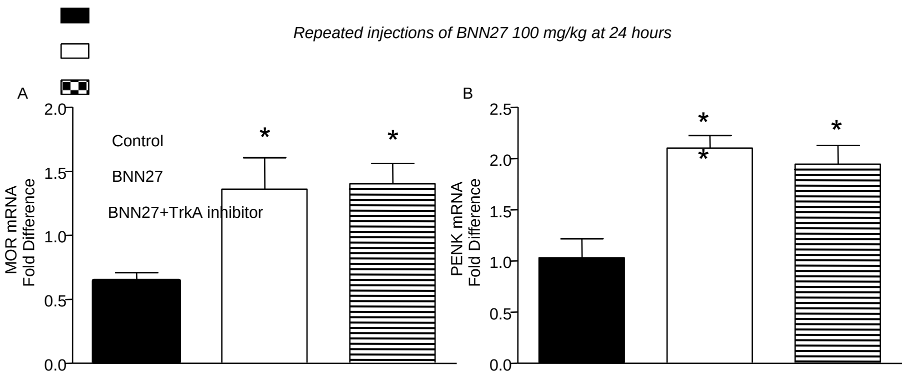

Figure S6: Administration of BNN27 100 mg/kg simultaneously with TrkA inhibitor did not affect μ-opioid receptor and PENK mRNA compared to mice treated only with BNN27
